# Navigating the Multiverse: a Hitchhiker’s guide to selecting harmonization methods for multimodal biomedical data

**DOI:** 10.1093/biomethods/bpaf028

**Published:** 2025-04-17

**Authors:** Murali Aadhitya Magateshvaren Saras, Mithun K Mitra, Sonika Tyagi

**Affiliations:** IITB-Monash Research Academy, Mumbai, Maharashtra 400076, India; Department of Physics, Indian Institute of Technology Bombay, Mumbai, Maharashtra 400076, India; School of Translational Medicine, Monash University, Melbourne, Victoria 3181, Australia; Department of Physics, Indian Institute of Technology Bombay, Mumbai, Maharashtra 400076, India; School of Translational Medicine, Monash University, Melbourne, Victoria 3181, Australia; School of Computing Technologies, RMIT University, Melbourne, Victoria 3001, Australia

**Keywords:** multimodal integration, feature representation, data integration, deep learning, digital health

## Abstract

The application of machine learning (ML) techniques in predictive modelling has greatly advanced our comprehension of biological systems. There is a notable shift in the trend towards integration methods that specifically target the simultaneous analysis of multiple modes or types of data, showcasing superior results compared to individual analyses. Despite the availability of diverse ML architectures for researchers interested in embracing a multimodal approach, the current literature lacks a comprehensive taxonomy that includes the pros and cons of these methods to guide the entire process. Closing this gap is imperative, necessitating the creation of a robust framework. This framework should not only categorize the diverse ML architectures suitable for multimodal analysis but also offer insights into their respective advantages and limitations. Additionally, such a framework can serve as a valuable guide for selecting an appropriate workflow for multimodal analysis. This comprehensive taxonomy would provide a clear guidance and support informed decision-making within the progressively intricate landscape of biomedical and clinical data analysis. This is an essential step towards advancing personalized medicine. The aims of the work are to comprehensively study and describe the harmonization processes that are performed and reported in the literature and present a working guide that would enable planning and selecting an appropriate integrative model. We present harmonization as a dual process of representation and integration, each with multiple methods and categories. The taxonomy of the various representation and integration methods are classified into six broad categories and detailed with the advantages, disadvantages and examples. A guide flowchart describing the step-by-step processes that are needed to adopt a multimodal approach is also presented along with examples and references. This review provides a thorough taxonomy of methods for harmonizing multimodal data and introduces a foundational 10-step guide for newcomers to implement a multimodal workflow.

## Introduction

The growth of biological and healthcare data, in terms of volume, velocity and variety, has been exponential and driven by technological advances in electronics, communication and infrastructure [1, 2]. Concurrently, there has been an increase in data analysis tools to understand and analyse the data. Progress in computational techniques, artificial intelligence (AI) and machine learning (ML) methods have been identified to contribute towards the analysis and interpretation better than traditional analytical methods [3, 4].

Data generated in the context of biological systems can manifest in various forms such as quantitative, qualitative or narrative; each has its subtypes, collectively referred to as a ‘modality’. These diverse modalities can capture several aspects of a biological system, such as nucleic acid and protein sequences [5], gene expression [6], and the biomolecular structure and its activity [7]. Other modalities include the epigenetic state and methylation information [8] of the genome, metabolites, and anatomic and phenotypic data.

Each data type has driven research towards elucidating the corresponding functional aspects to understand the system. Numerous studies using a single data modality have presented valuable additions to the literature in disease mapping, pathway and network elucidation [9–11]. However, a vast portion of the biological complexity still requires an explanation, an ongoing challenge for the research community.

Different modalities capture different aspects of the system. Thus, integrating them provides a comprehensive multi-view understanding of a biological system [4, 12]. Combining multiple types of omics data or a ‘multiomics’ approach to study biological systems has gained momentum lately due to their demonstrated superiority over single-omics approaches [3, 13–16]. Furthermore, healthcare data is integrated with omics datasets to reveal their interconnections, providing a comprehensive 360-degree view of an individual’s condition [17, 18]. Such studies have reported significantly validated and reliable results compared to independent analyses. Thus, integrating multiple modalities can reveal synergistic effects, where the combined information enhances the model’s overall performance beyond what individual modalities can achieve.

However, analysing multiple modalities together is challenging, given the heterogeneity of the data. Current research covers the merits of using general ML methods for the analysis of biological and clinical data [19, 20]. It is easier for experienced researchers in the domain to understand and implement hybrid methods and complex ML models. Still, the absence of adequate algorithmic information impedes interested researchers from fully grasping the process and implementing a workflow. Previous review articles on this subject relay information to mitigate data challenges but lack information on multimodal implementation [3, 21, 22]. A definitive explanation of the methods involved in a multimodal approach is missing.

Additionally, a gap persists in delineating between ‘integrated learning’ and ‘co-learning’ methods. While they are used synonymously, they indicate different concepts. Integrated learning refers to a broad, independent analysis of multiple modalities and linking the results to obtain a high-level overview. Co-learning, which we refer to as harmonization in the current review, allows cross-talk between elements across modalities, enabling to understand the relationships between modalities. Harmonization aims to elucidate the low-level relationship between features of different modalities [15]. Articles incorporating ‘integration’ as part of their pipeline do not necessarily perform a harmonization process. Instead, they focus on the correlation between individual data type analysis [13]. The effect of an analysis using multiple modalities is not adequately captured by methods that do not harmonize the features. A co-learning set-up is distinct from an individual analysis since it necessitates a fusion of features.

**Figure 1.**
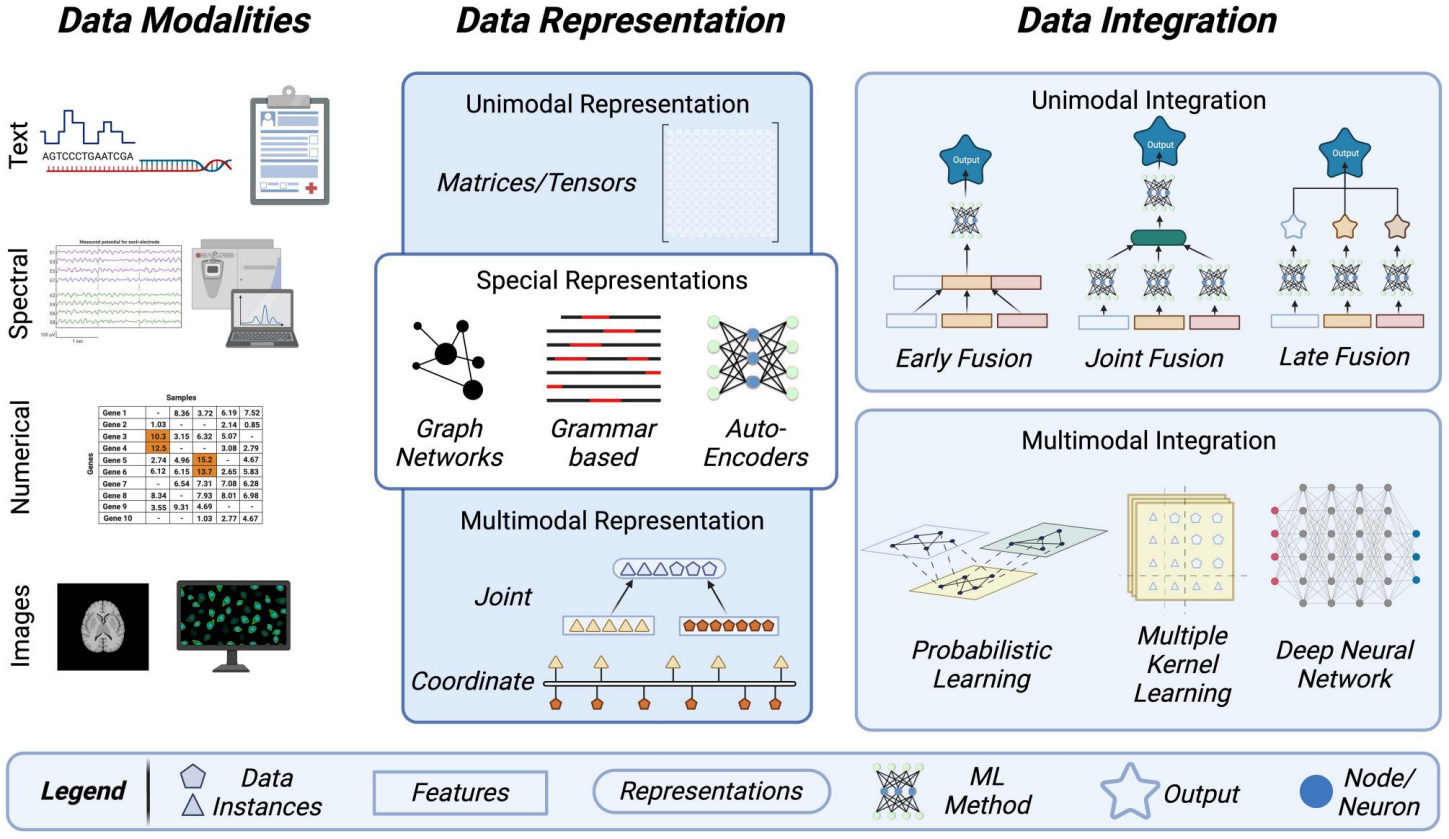
An illustration briefly depicting the broad categories of data modalities and the representation and integration methods used in a multimodal harmonization analysis. The representation methods are split into three groups based on the number and type of datasets. The integration methods are split based on the type of fusion performed. Made with BioRender

In summary, this literature review addresses the aforementioned gaps by offering insights into data modalities, challenges encountered in data, the components involved in a multimodal workflow (Fig. 1), and a beginner’s guide to multimodal analysis (Fig. 4).

## Data modalities

In the following sections, we describe the various data types, technical hurdles, and harmonisable methodologies gleaned from the literature we have examined.

### Typical study designs in biomedical and clinical studies

All data generated are usually based on a study or an objective with a focused rationale. Data for an analysis can be collected by the researcher (primary), or collaborated from other sources (secondary), and it can scale from small sample sizes within a local institution to large sample sizes from a global community [23]. The richness of information in collected or generated data depends on its type, determining the range of possible analyses [24] (Fig. 4).

A static study design acquires data as a ‘snapshot’, that is, collected at a point in time. Case series refer to static data collected from positive-group criteria within a population subgroup, while case-control studies include a negative dataset (controls) for comparison. On the other hand, cohort studies and randomized controlled trials sample data over a period of time, capturing the dynamic nature of a biological system, which allows for a realistic investigation. However, they are resource-intensive methods that must be maintained regularly, and constant follow-up with the subjects considered in the study is crucial. A single type of data is commonly collected from a sample population. Some datasets containing different types of data are generated from the same set of samples, and this is referred to as ‘multi-view data’. Multi-view data offers higher association in inferring correlations between data types because the same samples are used [25].

Many efforts are being taken to enhance the data collection methods and accessibility across various domains, such as in cancer (The Cancer Genome Atlas, https://www.cancer.gov/ccg/) and preterm birth [26]. Current literature predominantly reports on results based on single, static datasets. Correlation studies use multiple datasets to support conclusions through overlapping results [27]. Only a few methods take a complementary approach between modalities [13, 28].

### Common data modalities studied in the literature

Data can be collected in forms such as text, numbers, and multimedia. Based on the sources, they can be classified as ‘biological’ data and ‘health’ data [2]. We refer to ‘biological data’ as information from high-throughput experiments such as sequencing, expression profiling, microscopic imaging and the vast literature corpus for functional annotation. It also includes metadata related to samples, experimental design, assay protocols and technologies. ‘Health’ sources refer to data primarily collected through healthcare providers in digital forms. This data contains an individual’s valuable health and medical history and is stored as time-stamped electronic medical records (EMR). EMR data displays significant structural diversity since it can be structured data, including vital signs and pathology measurements organized in tabular formats, or presented as unstructured data, consisting of clinical notes, images, and documents. Table 1 describes the different modalities that stem from clinical and biomedical sources.

**Table 1.** The four distinct modalities of biological and clinical data investigated in this study are listed and categorized based on their sources and the features targeted for modelling.

Type	Data source	Features of interest	Common formats	Reference
Text	DNA, RNA, and Protein Sequence	Sequence order and motifs	FASTA, FASTQ	[37, 38]
	GWAS Data	Genetic variants	VCF, CSV, TSV	[39]
	Clinical Notes	Correlated medical terms and phrases	TXT	[40]
Spectral and signal	X-Ray crystallography, NMR, Mass Spectroscopy	Structural composition and identified functional groups, topology	SPC	[31]
	Audio signals	Speech to text patterns	WAV, FLAC, M4A, MP3	[16]
	Biomolecular profiles (Lipids, Metabolites, Nucleic acids, Proteins)	Expression levels of biomolecules	CSV, TSV	[19]
Numerical	EMR (Vitals, Lab measurements)	Health factors, trends and trajectories	CSV, TXT, Database Schema	[40]
	Interaction Networks (Diseases, drugs, genes, proteins)	Regulatory and Functional relationships	SIF	[41]
Images	EMR (CT, X-ray, Ultrasound)	Patterns and localizations	PNG, TIFF, JPG, DICOM	[42]
	Cell Imaging	Patterns and localizations	PNG, TIFF, JPG, DICOM	[17]

#### Text modality

Text as a modality comprises various types, encompassing narrative and sequence forms of data. Sequence data stemming from biological macromolecules such as DNA, RNA, and proteins describe and define the relationship between the genotype and the phenotype of an organism. Differences in sequences among groups differing in demography or phenotype are represented as single nucleotide polymorphism (SNP) or small insertions and deletions of DNA bases and are generated by genome-wide association studies (GWAS) [29]. Information on motifs, interaction networks, and annotations about biomolecules, drugs, and diseases belong to this class. Healthcare data, such as EMR, contain unstructured clinical notes and prescriptions manually entered by medical practitioners, which are included in the text category of datasets [30].

#### Spectral and signal modality

Spectral data, typically acquired through mass spectrometry experiments to study protein molecules and metabolites, provides detailed insights into the structural composition, constitution and organization of the molecules under investigation [10, 31]. ML analysis of spectral data involves features representing three-dimensional conformations and spatial relationships of molecules, enabling classification based on functional groups and elements [31, 32]. Proteomics, examining proteins through expression, functional relationships, and structural information, includes investigations into protein folding and structural orientations using methods such as NMR and X-ray crystallography [33]. Structural metabolomics stores the structural data collected from metabolites. Healthcare data in spectral form includes time-dependent electroencephalogram (EEG) and electrocardiogram (ECG) analysed with signal processing methods [34, 35]. Audio data, such as voice notes, undergoes analysis using appropriate methods after feature extraction [36].

#### Numerical modality

A numerical form of biomedical data can be from any quantifiable assay, broadly called ‘omics’ data. Transcriptomics represents digital counts of identified expressed transcript molecules, available as a two-dimensional (2D) matrix of genes/transcripts and samples. Similarly, proteomic, lipidomic and metabolomic counts data portray the expression levels of proteins, lipids or metabolite molecules as a 2D data matrix. This modal information allows understanding individual differences regarding genetic expression and linking related biological pathways. EMR readings document vital and pathological parameters such as heart rate, weight, height, age, and blood pressure as numerical time-series data. Frequent time-stamped EMRs enable longitudinal analysis, capturing changes in the recorded values over time [43].

#### Image modality

This modality encompasses visual information, including images and videos. Microscopy and cell imaging data are often analysed for morphology studies, protein localization and DNA tagging [44]. Manual image analysis methods include segmentation tasks to identify regions of interest and cell morphology assessment [45]. Videos are also considered under this modality because each frame can be considered an image for processing but in a time-dependent manner [36]. Cell movement and tracking studies creating animated clips from multiple fluorescence-tagged cell images are a modality of this category. Additionally, X-rays, CT, and MRI images from EMRs supporting non-invasive diagnosis fall in this category.

### Common challenges associated with biomedical data

Datasets require extensive preprocessing due to incompleteness and imperfections before analysis [46]. Key challenges include high dimensionality, heterogeneity, missing data, class imbalances, bias, and accessibility.

Complex high-dimensional data, characterized by large features and file sizes, require extensive computational resources for understanding the variables [47]. Addressing the ‘p≫n’ problem, considering the ratio of available samples (n) to features (p), is crucial to prevent specific features from being overlooked in small sample groups [15, 47].

Bias refers to a variety of imbalances found within a dataset and can lead to an unfair interpretation of results. Bias can manifest in various forms, such as representation bias or class imbalance, measurement bias (due to incorrect or unrelated values), aggregation bias (when models are applied to new datasets with a mutually exclusive relationship to training samples) and evaluation bias (when generic models are used as benchmarks for targeted datasets) [48]. Comparative analytical methods utilize representative datasets, subsets of the population with samples from distinct groups like case and control. It is crucial for groups to have samples in a comparable and an equivalent number to understand the true differences.

Missing data significantly undermines the strength of a computational analysis. Missingness can be within a modality (missing data points) or an entire modality. Incomplete or missing data within a modality can be due to a variety of reasons, such as irregular data collection processes, lack of information, technical and experimental errors [49]. In multi-view datasets, there might be missing modalities for a few samples [50].

Heterogeneity refers to the variety that exists within and across modalities. Within a modality, the data collected across variables can vary in terms of scale, distribution and recorded value, such as discrete, continuous, categories and intervals, due to non-standardized procedures. For example, clinical and genomic data cannot be directly compared and analysed, requiring methods to address heterogeneity.

These challenges obstruct the potential in any analysis, but the problem is exacerbated when multiple datasets are involved in a multimodal set-up [51]. Multimodal methods are affected by coherence between dataset sets (due to heterogeneity and missing data), accessibility and computational resources (due to high-dimensional datasets and bias).

Most of the challenges observed in raw data can be attenuated using pre-processing methods. Data preprocessing allows one to check, sort, and select data points so that informed decisions can be taken to handle samples with anomalies and poor quality. Cleaned but large datasets can be converted to latent values to reduce computational load. Missingness in data and modalities are handled by imputation during preprocessing and advanced ML architectures [52–56]. The lack of data standardization between multiple collection sites poses a challenge for seamless data harmonization [57] and requires specific preprocessing for different sources. Hence, more data-driven approaches may be adopted in different scenarios [57].

Biomedical and health data containing personal and sensitive information are restricted for global access, which limits the extent and scope of analysis. Implementing ethical and legal data practices, both nationally and internationally, is crucial for easing the data sharing process [58]. These practices establish a structured and transparent approach to handling data, creating an environment conducive to sharing valuable information.

The following sections on data representation elaborate on methods that can effectively handle data heterogeneity and high-dimensional data.

### Multimodal data harmonization: an investigation of the taxonomy

Multimodal analytical methods aim to combine information from multiple modalities towards one or many of the following goals: (i) Explain a biological phenomenon or phenotype through overlapping results. (ii) Account for and impute missing data in one modality through another linked dataset/modality. (iii) Condense the high-dimensional, sparse and noisy data to a low-dimensional latent representation.

The fundamental difference between an unimodal and a multimodal analysis is the number of different modalities used. The complete dataset can be directly fed into ML architecture for an unimodal analysis, but a multimodal approach requires the fusion of features from multiple datasets. The process of merging and modelling features can be classified under ‘representation’ and ‘integration’. The choice of method varies depending on the task to be achieved and the dataset combinations.

### Data representation

As discussed, biomedical and health data is generated in many forms (Table 1). Data representation methods make data amendable for modelling. This may require a data transformation step that converts diverse data types into machine-processable formats such as vectors, matrices, or tensors. Vectors are one-dimensional representations of numerical values, while matrices and tensors hold data in 2-dimensional and multidimensional scales. These methods keep track of relationships between elements of each modality via predefined rules, facilitate feature extraction by using relevant information, and help map data from one modality to another. Importantly, the representation methods can be modified to suit the study conducted [20]. In this context, we have classified three groups of data representation approaches.

#### Unimodal data representation

Unimodal data representation involves using a single mode or source of information to represent features. Each modality qualifies as an unimodal representation when independently transformed into a numerical format through an encoding or embedding approach.

Encoding involves converting original data into a numerical format, whereas embedding refers to portraying the original data in a vector space that incorporates semantic information. The information from biological sequence and text can be encoded by converting them to a numerical representation based on composition (tallying frequency of words/monomers), K-mers (segmenting a biological sequence as a window of ‘k’ letters) and distribution (percentage of occurrence of each monomer within user-defined ranges) of the sequence [59]. K-mers serve as the counterparts to n-grams or tokens in NLP methods, and ongoing efforts are focused on developing more advanced, data-driven approaches to derive them from sequential data [60]. Many tools have been created to embed text data into a numerical representation, such as word2vec [61] or [62], which preserves the order of information and local neighbouring relationships.

Numerical data obtained as-is or in other formats is commonly represented as matrices for analysis purposes. Time-series information is represented as tensors, where the data is nested within matrices and extending in dimensions to include the temporal relationship [63]. Similarly, image data is converted to a matrix representation by splitting a digital pixel into a numerical value between 0 and 255 for constituent colours. Furthermore, spectral information from biological mass spectrometry studies generates coordinate data and is represented as a matrix [64].

Unimodal representation is the fundamental way to proceed with any ML analysis (Table 2). The complete set of features obtained through representation methods can vary in size and dimension depending on the dataset. To alleviate the computational load and resources during modelling, feature selection and feature reduction methods reduce the representation into a smaller latent space portraying the complete dataset, which is used for analysis. There are multiple feature reduction methods, such as the Principal Component Analysis (PCA), Joint Non-negative Matrix Factorization (Joint NMF), and Autoencoders. Wrapper methods (forward, backwards, and stepwise selection), Filter methods (ANOVA, Pearson correlation, variance thresholding), and embedded methods (Lasso, Ridge, Decision Tree) are all part of feature selection techniques [22].

**Table 2.** Representation methods detailed with their advantages and disadvantages.

Representation		Advantages	Disadvantages
Unimodal		Simple; Interpretable; Allows interdepencies	Cannot capture contextual information; Susceptible to noise or biases; High feature sizes
Multimodal	Joint	Combines features to common space; Controls modality size effects; Allows interdependencies; Interpretable; Reduces dimensions;	Requires tailored architecture; Relies on meaningful cross-modal relationships;
	Coordinate	Aligns features to common space; Controls modality size effects; Allows interdependencies; Interpretable; Reduces dimensions; Imputes information; Captures contextual Information	Requires common axis for representation; representation depends on quality and definition of common space
Special	AEs	Creates latent representations; Controls modality size effects; Allows interdependecies; Reduces dimensions; Low susceptibility to noise	Low interpretability; Computationally expensive;
	Graph	Represents qualitative and quantitative; Interpretable; scales with feature size; covers global information	Requires domain-specific knowledge for feature extraction; Complex algorithms needed for irregular structures and dynamic graphs; Susceptible to missing information
	Grammar	Applies for text modality; Captures patterns and semantic information; Reduces dimensions; Interpretable;	Affected by ambiguity and complex language constructs; Computationally expensive; Large datasets needed for processing; Susceptible to missing information

#### Multimodal representation

**Figure 2.**
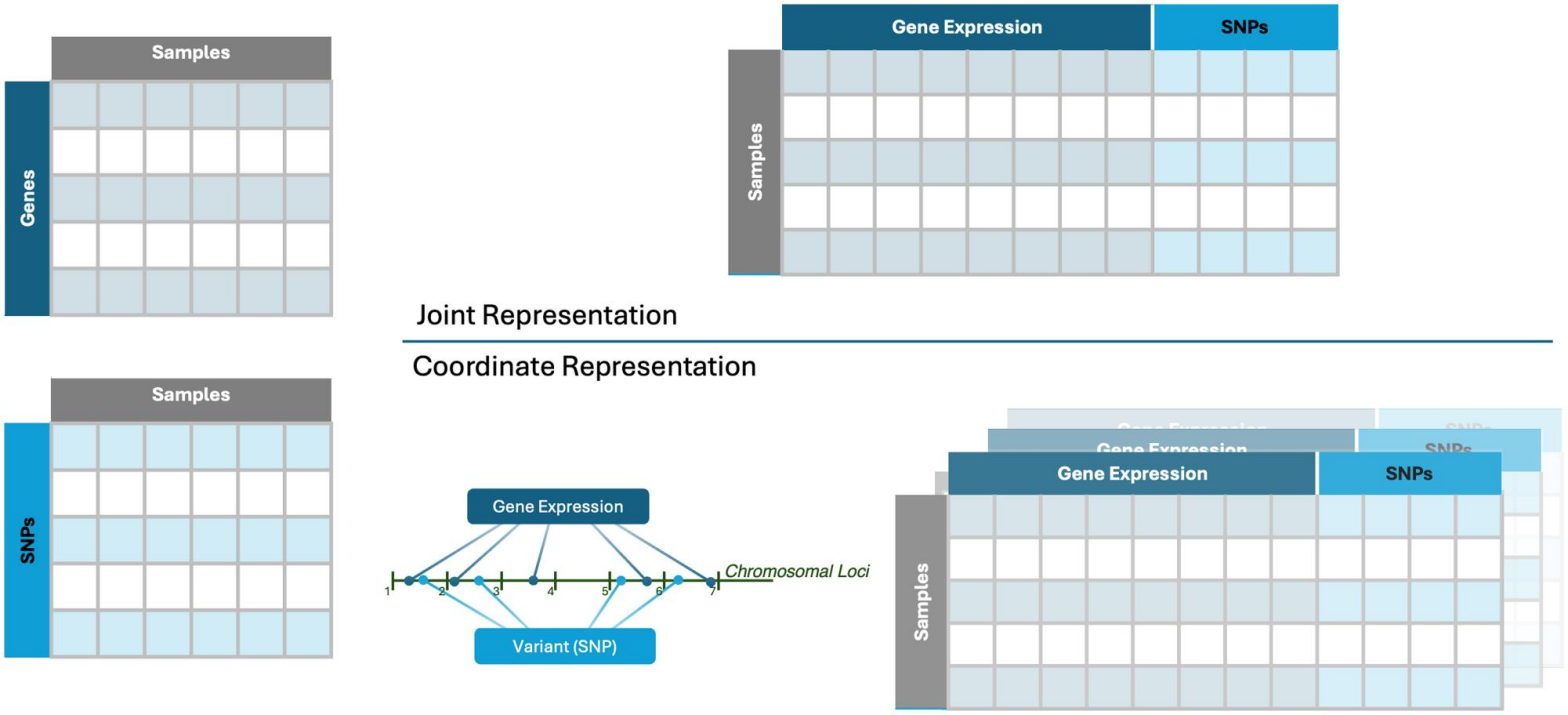
Illustration of a multimodal representation for a multiomics dataset. In this illustration, the joint representation is a direct concatenation of the modalities while coordinate representation aligns them against a common axis of chromosomal position and maps the data correspondingly

Multimodal data representation involves using multiple modes or sources of information to represent data. A multimodal representation fuses multiple unimodal representations together onto a shared feature space (joint) or co-represents the features from the different datasets (coordinate) (Fig. 2).

Each modality is condensed in a joint multimodal representation, and the defining features selected are concatenated to form a single collective representation (Fig. 2). The ratio of features from each modality contributing to the concatenated representation is maintained uniformly. This prevents modalities with fewer features from being overwhelmed by modalities with large dimensions. Zhao *et al.* describe the application of joint representation in two publications using image data and clinical information [65, 42]. They merge representations of image data (CT scans) and clinical information in different ratios and predict lymph node (LN) metastasis [65]. In a subsequent publication focusing on the same diagnosis, they introduce the 3M-CN architecture that utilizes a ‘refine layer’ to predict LN metastasis [42]. The refine layer is a concatenation of key features identified from clinical information and processed 3D images.

Coordinated representations reduce and present the features within each modality individually but link them towards the same meaning over a common coordinate space (Fig. 2). Trajectory inference or pseudotemporal ordering is a method to classify the different stages of the same cell type along an axis representing evolution [66]. Pseudotime ordering is an excellent example of coordinate representations, where data from single-cell experiments are projected onto an evolutionary axis [66]. The relationships established with identified patterns and domain knowledge help associate the features. MATCHER is a tool that has depicted imputation and correlation between modalities using a coordinate representation [28]. The manifold alignment method used in this tool achieved this task by representing data in low dimensions called a manifold and aligning them in a common space (alignment) [67].

Multimodal representation methods empower ML architectures to investigate the interplay between features across diverse modalities (Table 2). The entities in a biological system interact with each other in varied ways. Hence, the ratio of representations in the shared space as a parameter also affects the results of a multimodal analysis [65]. Coordinate representations become more complex than joint representations when there is no common ground to link the features.

#### Special representations

Special approaches represent data non-conventionally through a generative or rule-based approach. These are not mutually exclusive to the previous two categories but process one or more source modalities differently to generate a representation. Generative representations learn the underlying patterns and structure of the data and are capable of generating new instances of data that are similar to the examples they were trained on. On the other hand, rule-based representations leverage formal rules and semantics to describe the features within a dataset.

##### Auto-encoders (AE)

AE methods compress the entire dataset into a compact set of dimensions through an ‘encoding’ process, eliminating any non-representative features. A ‘decoding’ process then reconstructs the original data using the condensed representation, validating the reduced feature space. The decoding layers are generative of the relationships between all the variables within the data, and hence, this method is classified under a generative representation approach.

Detlefsen *et al.* extensively explores AE-based representations, emphasizing the superior results achieved through the non-linear representation method in various tasks [68]. Zhang *et al.* introduce OmiEmbed as a multitasking framework, utilizing the low-dimensional latent space generated by AEs for downstream tasks like cancer classification and survival prediction [69]. AE representations also find applications in gene identification and cancer detection using expression data [70] and predicting carcinoma primary sites through DNA methylation data [71].

**Figure 3.**
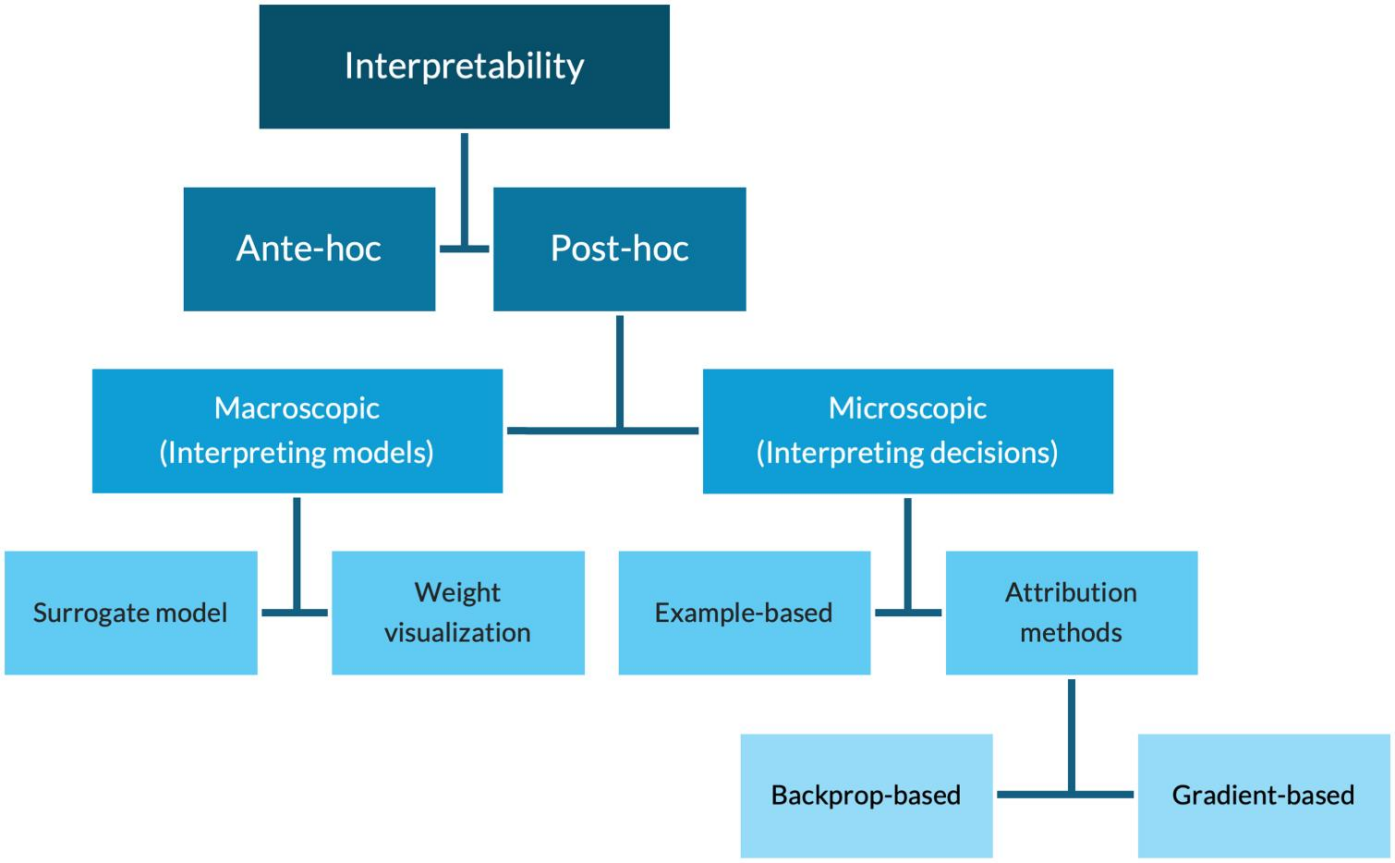
A flowchart describing interpretability methods for ML algorithms.

The encoding process in AEs can incorporate any type of model, such as a fully connected neural network (FCNN) or convolutional neural network (CNN), to generate the latent space [69]. Multiple modalities can also be combined at the input to generate a joint latent space [72]. This allows to generate different variants of the latent space and increases the choices available to work with. The validation by the decoding process makes the latent space devoid of errors or data misrepresentation. However, the interpretability of AEs is low, and requires special methods for interpretation (Table 2, Fig. 3).

##### Graph-based

Graph representations portray relationships between different entities as a network by considering them as ‘nodes’, and the relationship is depicted using ‘edges’. The edge values denote characteristics like similarity, interaction, and affinity between nodes based on the data. Graphs can be generated from experimental data (e.g. omics), theory and literature (e.g. disease networks) to represent qualitative and quantitative information. Clinical information about diseases and drugs can also be represented as graphs with links depicting common pathologies and targets. Intramodal networks describe relationships between identical node types (for example, protein-protein), while intermodal networks depict links between distinct node types [41]. Omics modalities such as genome, lipidome, metabolome, proteome, and transcriptome can be fused with environment and EMR modalities and represented as a Heterogeneous Multi-layer Network (HMLN) [73].

Specific ML architectures are devised to best use a graph network representation to map and predict links between nodes. Graph Convolutional Networks (GCN) create features from local graph structures to learn, and they can be scaled based on the number of interactions to represent complex relationships [74]. Graph Attention Networks (GAT) incorporate attention mechanisms to overcome structural overfitting for higher-order GCNs [75]. Ghorbani *et al.* presents the MGCN architecture, which implements graph representations to consolidate multilevel data [76]. Sparse data matrices are easily translatable into graphs, as they efficiently condense large-dimensional data to relevant nodes. The methods are highly sensitive to missing and unseen information but excel at discovering links within datasets (Table 2).

##### Grammar-based

Grammar-based or semantic methods rely on a predetermined, ordered set of ‘vocabulary’ to generate a representation, usually for a text-based modality. High-level patterns observed in the modality are identified, and the complete dataset is represented based on the patterns discovered through a feature generation procedure. Additionally, data can either be embedded based on the provided dataset or referenced from the complete knowledge bank. Dictionary-based embedding methods create embeddings for the complete corpus, and the available data is represented based on the closest relationship from the complete dictionary [15].

Grammar-based representations have been used to embed the motif information from sequences with the domain knowledge to depict the functionally connected regulatory regions [77]. Tyagi *et al.* used grammar-based representations to model the syntactic and semantic rule of RNA folding and used context-free grammars (CFG) to generate sequences and parse their structures [37]. Andikos *et al.* created Knotify [38], a tool to predict RNA pseudoknots using CFG. Onokpasa *et al.* assert that CFG representations improve compression ratios of RNA sequences and structures [78]. Recently, genome language modelling (GLM) is emerging as an application in the domain of genomics. Here, the sequences (a text modality) are considered as a ‘language’ with semantics and syntax and modelled to understand and predict biological functions [79–82].

Although grammar-based representations are powerful in capturing structured information, they may face challenges in handling the inherent ambiguity and variability in real-world data. They require large amounts of data and computing power to process and generate the rule-based representation [15].

In a few cases, ML methods do not differentiate between the representation steps and model training. For instance, dense neural networks do not explicitly have a joint representation stage. They are directly processed to learn the features of the data and train the model.

### Fusion of data modalities: integrating multifaceted information

Data fusion methods harmonize the different data modalities available for tasks such as clustering, regression, or classification, utilizing the representations from the methods discussed above. Different modalities can be harmonized using two broad ML approaches: Unimodal learning and Multimodal learning.

#### Unimodal learning

In a multimodal integration process, this approach treats each modality individually, and each modality requires dedicated processing before fusion occurs. Here, three categories of unimodal learning-based integration exist based on how the features from different modalities are fused: (i) Early, (ii) Late, and (iii) Joint integration.

##### Early fusion

Early fusion methods describe ML architectures that concatenate feature representations from multiple modalities at the input stage for modelling. The data is minimally processed, primarily to resolve heterogeneity, and samples are removed if imputation is impossible for missing data. Since the data is fused at the input stage, only one unified model is needed for training and inference. This reduces the complexity of managing multiple models or sub-networks for each modality. This method disregards prior selection bias and allows us to investigate all features across modalities. It is time-consuming and computationally expensive to process the complex combinations of all features from modalities [2].

The benefits of using early integration methods are discussed and reported by Barnum *et al.* [83]. They assert that using immediate fusion techniques to merge modalities before feeding them into a model works better by integrating the lowest statistical correlations between input features. Banerjee *et al.* describe the PERFORM algorithm, utilizing EMR data represented as temporal vectors, to assess its prediction performance in diagnosing acute pulmonary embolism (PE) [40] with ElasticNet architecture [84].

In the case of early fusion, class imbalance and differing sample sizes across modalities can affect the contribution of individual datasets, potentially biasing the analysis. Moreover, as the number of harmonized modalities increases in early fusion methods, the interpretability of the model drastically reduces. The limited coherence among data modalities restricts their combined usage, creating challenges in achieving a unified representation (Table 3). For example, input data as a combination of metabolomics and chromatin accessibility data may hinder a unified representation. Chen *et al.* describe data agnostic and data specific methods, with choices of modalities that can be used for coherent analysis [14].

**Table 3.** A description of the integration methods and their advantages and disadvantages for a multimodal set-up.

Integration		Advantages	Disadvantages
Unimodal	Early	Requires dedicated processing for each modality separately; Can performs lower-level of statistical correlation between features; Disregards selection bias	Computationally complex and expensive; Prone to data loss as matching features across modalities are required. Requires tailored models for effectively modelling and interpreting features
	Joint	Mitigates heterogeneity during modelling; Reduced feature size due to representations eases computational load	Requires tailored models for modelling joint representations
	Late	Independent models for learning different modalities; Ignores representation bias	Does not allow for feature interaction
Multimodal	Probabilistic	Highly interpretable; Establishes links between entities across different modalities	Requires domain-specific multilayer network information; Susceptible to missing data
	Multiple kernel Learning	Models based on overlapping results; Applicable for non-linear relationships; Resistant to outliers; Interpretable	Susceptible to missing data
	DNNs	Uncovers hidden information without explicit rules; Utilizes complex architectures to understand the non-linear relationships between features and modalities	Low interpretability; Computationally complex and expensive

##### Late fusion

Late fusion methods analyse multiple modalities independently using a model that best fits its representations to a predicted output, and the outputs from each are aggregated towards a singular result or inference. Late integration is generally performed either by taking an aggregated average of the predicted probabilities (outputs) from each modality or passing all the predictions from each modality into an FCNN to process a final output.

Wang *et al.* presented MOGONET as a tool to classify cancer subtypes using three modalities: mRNA expression, methylation and miRNA expression [85]. A late fusion architecture was established using GCN to predict an initial class label and an FCNN to generate a final class label prediction. Luo *et al.* presented a modified version of MOGONET, called GRAMINet with GATs instead of GCNs [87]. Both examples pass the same combination of modalities through different architectures to learn a model for biomedical data classification. This is the advantage of using late fusion—flexibility to apply different algorithms to each modality for modelling. Huang *et al.* investigated a multimodal approach to predict PE and reported the results on seven different architectures, one early, two joint and four late fusion architectures [88]. The early fusion methods had the highest sensitivity, while the late ElasticNet architecture outperformed in all other metrics, such as accuracy, AUROC, specificity and positive predictive value.

Ensemble learning may be considered a variant of the late fusion model, where the outputs of multiple ML models are combined towards a final decision [88]. Late fusion focuses on combining outputs or representations after individual processing, whereas ensemble learning leverages the diversity of multiple models to improve overall predictive performance.

Late fusion methods do not directly allow for the interaction of features from multiple modalities. It enables training each modality with independent, unique models without interference from other data types. As a result, concerns about different dataset sizes, heterogeneous measurements, and model compatibility vanish (Table 3). The ability of late fusion methods to capture all information within each modality equally makes it the widely reported harmonization method in the literature.

##### Joint fusion

Joint fusion methods endeavour to extract a representation of all initial modalities and model them together to a predicted output. Direct concatenation methods, often used in early fusion, are not possible between modalities that have different quantities and may require a heavy preprocessing step. In late integration, the interdependency between features across modalities is ignored. Joint integration methods provide an advantage through the interaction of features from different modalities in the training phase, irrespective of the observed heterogeneity. The heterogeneity is mitigated since the feature representation and selection procedures reduce and unify the information numerically.

Joint integration methods have been explored using modalities such as CT scans, EMR, methylation, and expression data to achieve biomedical tasks of classification towards prognosis and diagnosis [42, 65, 85, 87]. Zhao *et al.* investigated the effect of different ratios of EMR features during an integrated analysis of CT images using the DensePriNet architecture [42]. Huang *et al.* propose a joint representation of CT images and clinical features in a ‘refine layer’ to predict an output that performs better in detecting pulmonary embolism in comparison to other models [28]. MATCHER utilizes a joint fusion method to interpolate instances based on the alignment of multiple modalities to the pseudotime scale [28]. Multi-view datasets are analysed with a joint representation and fusion to obtain linked information between modalities. MOMA integrates two modalities from the same sample set towards cancer classification [89], and iDeepViewLearn presents an algorithm for more than two modalities [90]. DeepIDA-GRU jointly integrates both time series and static data in their algorithm [91].

Joint methods are more complex than late fusion but easier to interpret than early fusion methods. Using representations rather than the raw features from each modality dramatically reduces the computational load compared to early fusion. However, joint harmonization methods are not data agnostic and may require carefully curated model designs (Table 3).

#### Multimodal learning or co-learning

Multimodal learning is designed to integrate and model the features from different modalities more comprehensively than unimodal learning. Joint fusions merge representations, but multimodal learning enables co-learning through a direct feed of all the features interacting at the lowest, complex levels. They can be classified into three distinct co-learning methods: probabilistic, multiple kernel learning and deep neural networks (DNNs).

##### Probabilistic models

Probabilistic methods use joint or conditional probabilities to build models that capture the relationships and dependencies between different modalities. These models are highly interpretable, allowing them to integrate expert knowledge in the fusion approach and allowing us to interpret the results better than other methods.

The random walk method uses probabilistic values to simulate a particle moving between nodes and layers in a network, establishing the relationships and links between the nodes [92]. MultiXRank module, published by Baptista *et al.*, is an example of the probabilistic method of integration using a multiplex network of intramodal and intermodal interactions (protein-protein interactions, gene multiplex and disease monoplex networks) [92]. Pio-Lopez *et al.* describe a use case of the random walk with restart architecture, wherein the method predicts long-distance gene-disease interactions using gene interaction network and disease similarity network data [93].

Probabilistic methods are applicable to any combination of modalities as long as they form a multiplex network. They rely heavily on theoretical knowledge to bridge relationships between elements of multiple domains and hence can be applied to data from any domain with multiplex and bipartite networks [93]. Since they are dependent on existing information, probabilistic models are influenced by missing and unseen data (Table 3).

##### Multiple Kernel learning

Kernels are linear classifiers that divide the data linearly using lenient boundaries, and a combined multitude of them assist in classifying non-linear heterogeneous data [94]. This method is implemented in support vector machines (SVM), a popular method to analyse complex data.

In multiple Kernel learning (MKL), different kernels are applied to each modality, and the combination of these kernels is learned to optimize the model’s overall performance. Liu *et al.* used SVM to model MRI datasets from multiple sources towards Alzheimer’s disease classification [95]. Lancktiet *et al.* predict the functions of yeast proteins using kernel-based learning [96]. Multiple matrices describing the protein data were used in the algorithm, and results were reported on the different combinations of kernels used to classify the proteins as per their functions [96].

Kernels can identify linear boundaries in datasets, making MKL a highly suitable method for classification tasks [97]. Kernels can be combined in different ways (sum, product) to generate new kernels. A combination of multiple kernels accounts for a better classifier than using a single kernel [98]. This method is resistant to outliers but is susceptible to missing data [97] (Table 3).

##### Deep neural networks

Deep neural integration methods are characterized by a substantial number of neurons and layers constituting neural networks with significant depth and complexity. DNNs utilize representations of different modalities to reduce the features and pass them through high-level, intricate architectures, which enable them to uncover hidden information within the datasets.

DNNs are extensively used to understand data at a microscopic level, especially in the biomedical domain. EMR data can be modelled with omics modalities to shed light on physical and phenotypic changes and their relationships across time. Zhu *et al.* address an ML model to fuse and learn time-series data, with the use of Stacked Sparse Auto-Encoder (SSAE) and Long Short-Term Memory (LSTM) architecture [63]. Zhang *et al.* have reported about OmiEmbed, a multitask deep-learning framework based on an autoencoder architecture [69]. AffinityNet, proposed by Ma *et al.*, uses k-nearest neighbours (kNN) attention pooling where the cluster representations of the data are processed as a GAT [99]. The method has asserted good performance for both labelled and unlabelled datasets.

DNNs are computationally expensive to perform due to their dense and complex architecture. They model data at high degrees of non-linearity, but the process becomes hard to decipher and elucidate directly (Table 3).

### Model selection

Different combinations of representation and integration methods can be used with different modalities to perform an analysis. An expanded list of various published ML tools integrating multiple types of modalities has been curated in Table 4. Model metrics help select the best model for testing and deployment. Models can be ranked across metrics such as accuracy, loss, f1 scores, false discovery rates, Matthews correlation coefficient scores (MCC) and Receiver operating characteristic (ROC) curve. The choice of the metric depends on the task performed and the data [100].

**Table 4.** Guide matrix providing examples from literature based on different combinations of modalities and the corresponding tasks, study design, representation, integration, model used, interpretability and reference.

Modalities Integrated	Representation	Integration	Study Design	Task	Model Used	Interpretability	Validation	Ref
EMR^*^	Unimodal	MKL	Static	Prediction	SVM	High	Public, Internal	[130]
	Joint	Joint	Time Series	Risk Prediction	EN	Low	In-house, External	[40]
Gene Sequence^*^	Grammar	–	Static	RNA Structure Prediction	CFG	High	Public, Internal	[37]
	Grammar	–	Static	RNA Structure Prediction	CFG	High	In-house, Internal	[38]
	Grammar	–	Static	RNA Structure Prediction	CFG	High	Public, Internal	[78]
Protein Sequence^*^	AE	–	Static	Clustering	VAE, ResNet, LSTM	Low	Public, Internal	[68]
Disease network, miRNA	Graph	Probabilistic	Static	Disease Prediction	Katz	High	Public, Internal	[131]
Image, EMR	Unimodal, Joint	Early, Joint, Late	Static	Classification	FCNN, CNN	Low	In-house, Internal	[87]
	Unimodal	Joint	Static	Classification	DDB	Low	In-house, Internal	[88]
	Joint	Joint	Static	Classification, Segmentaion	DB, CNN, FCNN	Low	In-house, External	[42]
Methylation, Image	Unimodal	Early	Static	Multi-variate Regression	GLM	High	In-house, Internal	[17]
Metabolomics, Proteomics	Unimodal	Late	Static	Differential Correlation	–	High	In-house, Internal	[132]
Trancriptomics, DNA-Protein interaction network	Unimodal	Late	Static	Differential Correlation	–	High	In-house, Internal	[133]
Transctiptomics, Methylation	Unimodal	Late	Static	Differential Correlation	–	High	Public, Internal	[134]
	AE	Joint	Static	Classification	AE, FCNN	Low	Public, Internal	[135]
	Graph, Joint	Late	Static	Classification	SNF	High	Public, Internal	[136]
	Joint	Joint	Static	Classification	Attention FCNN	Low	Public, Internal	[89]
	Joint	Joint	Static	Association	FCNN	Low	Public, Internal	[90]
Non-Bio	Graph	Probablistic	Static	Classification	GCN	Low	Public, Internal	[76]
	AE	Joint	Static	Classification, Regression	SSAE, LSTM	Low	Public, Internal	[63]
Proteomics, EMR	Joint	MKL	Static	Clasification	SVM	High	In-house, Internal	[18]
Mutation; Drugs	Unimodal	Joint, Late	Static	Drug Prediction	CNN	Low	Public, Internal	[39]
Gene Expression, Chromatin Accessibility	Coordinate	Late	Time Series	Cell Trajectory Construction	MA	High	Public, Internal	[28]
Metabolomics, Transcriptomics, Metagenomics	Joint	Joint	Static	Association, Classification	IDA	High	Public, Internal	[137]
	Joint	Joint	Series, Static	Classification	GRU, IDA	High	Public, Internal	[138]
Networks of Gene; Drug; Disease	Graph	Probablistic	Static	Link Prediction	RWR		Public, Internal	[93]
	Graph	Probabilistic	Static	Link Prediction	RWR	High	Public, Internal	[92]
Methylation, mRNA, miRNA	AE	DNN	Static	Classification	AE	Low	Public, Internal	[138]
	AE	Joint	Static	Classification, Survival Analysis	AE, FCNN, CNN	Low	Public, Internal	[88]
	Graph	Joint	Static	Classification	GCN	Low	Public, Internal	[85]
	Unimodal, Graph	Late	Static	Classification	GCN	Low	Public, Internal	[86]
Lipidomics; Metabolomics; Proteomics; Transcriptomics	Unimodal	Joint	Static	Drug Prediction	PLSDA		Public, Internal	[13]
Methylation; Gene Expression; Mutation; Copy Number Alteration; Clinical	AE	Joint, Late	Static	Survival Analysis	FCNN	Low	Public, Internal	[139]
Gene expression; Metabolomics; Proteomics; Cytokine measurements; Cytometric measurements; Microbiome	Unimodal	Late	Time Series	Multi-variate Estimation	EN	Low	In-house, Internal	[140]
Interaction networks of drug compound, pharmacologic class, gene of action, pathway, biological process, disease, side effect, symptom, and anatomy	Graph	Probabilistc	Static	Link Prediction	EN	High	Public, Internal	[73]

GLM, generalized linear models; VAE, variational autoencoders; CFG, context free grammar; DB, DenseBlock; CNN, convolutional neural network; GCN, graph convolutional network; SVM, support vector machines; SSAE, stacked sparse autoencoder; LSTM, long short-term memory; SNF, similarity network Fusion; RWR, random walk with Restart; MA, manifold Alignment; EN, ElasticNet; AE, autoencoders; FCNN, fully connected neural networks.

* Are all representation methods.

ML models have been considered as a black box, often undecipherable and complex to interpret. Interpretability in machine learning can be split as ante-hoc interpretability and post-hoc interpretability [101] (Fig. 3). Ante-hoc interpretability methods refer to the usage of interpretable models such as decision trees and regression to train on the data [102]. They explain the relationship between variables intrinsically and helps understand how the predictions are generated. Post-hoc methods use special methods to analyse and interpret complex models after training. This can be categorized as macroscopic (model-focused) or microscopic (input-focused). Macroscopic methods attempt to explain the representations learned by the model using simple surrogate models, or by visualizing weights of the model [103–105]. Surrogate model examples include decision trees, Shapley additive explanations (SHAP) [106] and local interpretable model-agnostic explanations (LIME) [107]. Visualization of weights from different levels of a model help interpret the patterns identified by a model [105]. On the other hand, microscopic methods focus on *why* and *how* a decision is made for a given input. This helps verify the model behaviour. Example-based method provides evidence for a decision by showing the influence of instances from the training dataset [108, 109]. Similarly, attribution methods improve interpretability for image-based analyses by highlighting the features responsible for the models’ decision (by back-propagating node importance or smoothening gradients) [105, 110–112].

There is often a trade-off between models with low-performance, high interpretability (decision trees, linear regression) versus high performance, low interpretability (DNNs) since simple models cannot learn complex relationships [107]. However, many new methods have comparable or higher performance scores to complex architectures while also providing interpretability [49, 113, 114].

## Guidelines for model selection

**Figure 4.**
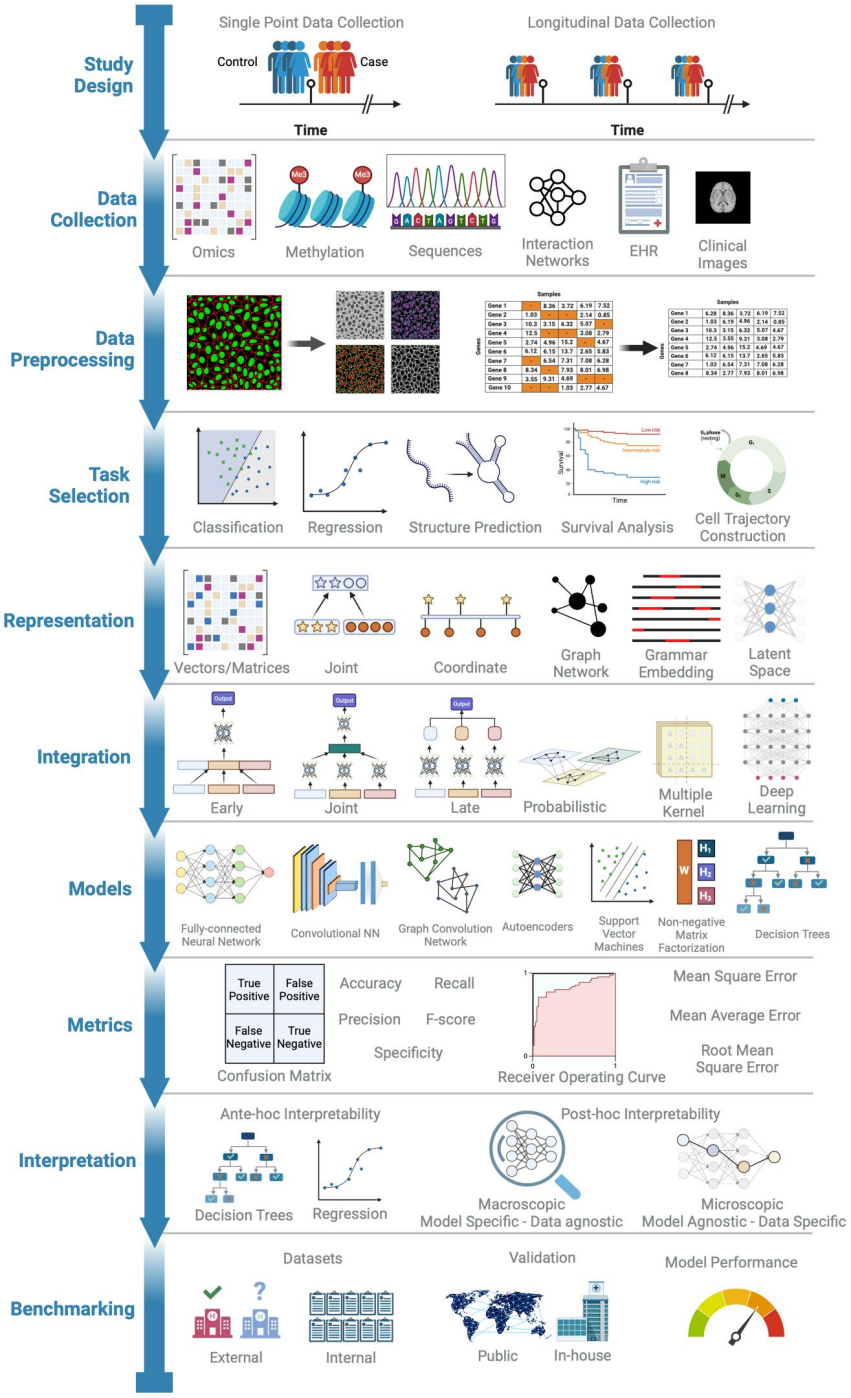
A 10 step guide flowchart that describes the process and order of execution to perform a multimodal integration. The titles on the left of the timeline describe the task order. The illustrations on the right are representative examples of different methods under each category. Made with BioRender

We propose ten recommendations for initiating a multimodal harmonization analysis (Fig. 4). Articulated objectives and aims are essential before initiating the harmonization analysis. These objectives will guide subsequent data collection, representation, and model selection steps.

1. **Tailor study design to objectives:** Tailor the study design to the defined objectives, taking into consideration the scale of the study and available resources. Ensure effective study design for sample identification and data collection that aligns with the study’s goals [115, 116].
2. **Implement optimized experimental protocols:** The data are either already available or generated through new experiments. Employ optimized protocols such as the FAIR Principles (Findable, Accessible, Interoperable, Reusable) for data and metadata collection strategies, ensuring consistency and reliability [117]. Here, both the study and software design protocols form the foundation for subsequent analysis steps and contribute to the quality of research outcome [118].
3. **Interoperable global sharing:** Digitize all collected data to facilitate analysis and global data sharing through repositories and databanks. Adopting interoperable data and metadata standards enhances data sharing and harmonization. It is a crucial step for collaborative research efforts and ensures data accessibility for future studies [117, 119].
4. **Modality identification and data preprocessing:** Classify the collected data into the broad modalities discussed here and systematically process the data individually to create a working subset free of artefacts and low-quality elements. Care must be taken during preprocessing to ensure minimum signal loss, and more data-driven approaches as opposed to ad-hoc rules must be used to discard uninformative data points. The analysis and results can vary depending on the level of preprocessing done [120, 57].
5. **Task selection:** Analysis of the target variable depends on the task selection, and it should be guided by the study’s goals. Choose tasks that reflect the aims and are applicable to the dataset.
6. **Choose feature representation and integration methods wisely:** The choice of representation or integration methods influences each other. We recommend selecting them according to the type of data, the number of modalities, the level of harmonization, and the coherence of the modality. Early and joint learning can be employed for linked modalities and late fusion for dissimilar data types. Recognize that a one-size-fits-all approach is impractical, and tailored methods may be needed for different tasks (Tables 2 and 3).
7. **Navigate model selection complexity:** Different ML models can be employed for the same harmonization set-up. Employ different models of varying complexity to assess the data and evaluate their performance using appropriate metrics (Table 4).
8. **Model performance metrics:** Select model performance metrics corresponding to the task to compare and choose the optimal model. Provide an explanation of the metrics used and their relevance to the task [121–125].
9. **Prioritize interpretable models:** Prioritize using interpretable models, either intrinsic or through post-hoc interpretation. Especially in clinical settings, understanding how a model arrives at conclusions enhances trust and reliability [102].
10. **Validate and Benchmark models:** Validate models on different datasets and sources to ensure robustness and generalizability. Benchmark models against state-of-the-art approaches and external datasets to mitigate aggregation and evaluation biases [126, 127].

Overall, these recommendations must be adapted to the specific context and goals of any multimodal harmonization analysis.

## Discussion

### Lack of comprehensive reviews

The article highlights a noticeable gap in the existing literature regarding comprehensive explanations of workflow and procedures for integrating biomedical multimodal data. Multiple reviews for machine learning strategies to process multimodal data are available, but there is a deficit of articles relating them to biological and clinical data. A predominant part of the research literature presents results with information from a single modality.

The concept of co-analysis, or more aptly, ‘co-learning’, is missed. There is a lack of clarity on how to effectively integrate data from disparate sources at the lowest item level to extract holistic knowledge. This review suggests methods that can be used to perform multimodal fusion.

### Diverse taxonomies in multimodal analysis

We highlight the various data types and the analysis methods available under a limited set of taxonomic categorizations. Biomedical multimodal data from the same sample set is now routinely available from various research and development activities and healthcare. This classification of data types from biological and clinical sources allows one to identify methods that will suit the analysis of specific combinations and evaluate the advantages of each.

In the context of multimodal analysis, there is a distinction between the representation and integration steps and unimodal analysis. We describe data harmonization as a split of representation and integration methods, each with six distinct categories. Most steps are similar to an unimodal analysis, and the distinction in a multimodal analysis arises in the representation and the integration steps. The representation methods proposed emphasize the features within the data. The various types of representation methods are key to uniformly presenting the multimodal data prior to an analysis. Integration techniques describe the various methods to feed the data into ML architectures.

### Framework and model suggestions for biomedical data combinations

There is a need for a structured framework or guideline to facilitate the harmonization process for multimodal data. The article addresses this gap by presenting the first guideline framework towards a data harmonization process and providing a complete workflow. The recommended procedure consists of 10 steps to plan through towards a multimodal analysis.

To assist those undertaking harmonization for the first time, we present a guide matrix showcasing examples from published literature, illustrating different combinations of data modalities. The combinations between the representation and integration methods are presented as a non-exhaustive list in Table 4. Existing studies show that different choices can yield different results when using the same datasets [87]. The diverse taxonomies outlined in this paper can assist in understanding the significance of choosing an appropriate integration model for analysis, considering the concern related to biomedical data and model challenges (Tables 2 and 3).

### Future focus for harmonizable models

The article acknowledges the challenges related to data and model selection in the context of multimodal analysis. Data-related challenges and model-related challenges both arise when implementing a multimodal analysis. Missing data and modalities are the most prominent factor in hindering a multimodal analysis. Many studies present methods to handle missing data points in modalities [56, 128] and also missing modalities within samples [129, 55]. Missing data patterns are shown to contain informative features and to help in modelling datasets [50]. In addition to the challenges related to biomedical data, concerns about data acquisition and maintenance also require attention. The quality of biomedical data collected needs to be maintained, with appropriate measures taken to de-identify the data and global sharing. A vast majority of the published literature on biomedical multimodal analysis focuses on the model metrics and parameter scores. However, due focus should be given to the model interpretability as well. Multimodal analysis with complex architectures may yield high performance scores, but they cannot be used to understand the biological and clinical data if the models are not interpretable. Many of these models offer interpretability- important edges from graph networks, tokens from grammar-based, weights from attention, and ranked kernels are a few examples that can be linked to the data from the model. Interpretable models are needed to understand the process, especially with biomedical data, and to relate to further procedures, such as diagnosis and intervention strategies. Tables 2 and 3 summarize methods that can mitigate the challenges of missing data and interpretability. Examples provided in Table 4 detail the level of interpretability of different ML models.

### Innovations in integrative models

Methods for integrating multiple modalities are being constantly investigated, and many methods have been published (Table 4). For example, the concept of attention in neural networks was first published in 2017, and multiple complex architectures have been developed with attention. With the increase in data, it is expected that methods will be developed to target granularity while maintaining generalisability for an analysis. Currently, a trend towards the use of large language models (LLMs) in the domain of biomedical research is increasing with the focus to create a generic model capable of a multitude of tasks. While LLMs can be finetuned and optimized towards specialized tasks, they are extremely data-dependent, have little interpretability and require high compute power for implementation and analysis. This prevents them from being deployed ubiquitously. The gap between the usage of high-performance models to interpretable models is decreasing, but data complexity still keeps them apart from being universally substituted.

## Conclusion

The article highlights a significant gap in existing literature regarding the integration of multimodal data, noting a lack of comprehensive explanations and holistic views in current research. While recognising the transformative potential of multimodal integration, it emphasizes the need for clarity on effectively integrating disparate data sources to extract comprehensive knowledge. Acknowledging challenges in data and model selection, the article proposes using diverse taxonomies to aid integration model selection. Addressing the distinction between unimodal and multimodal analysis, the article provides insights into representation and integration methods for multimodal data. Furthermore, it underscores the need for a structured framework to facilitate harmonization, presenting the first guideline framework and workflow. Additionally, it aims to assist researchers new to harmonization by offering a guide matrix featuring examples from published literature, aiding in selecting appropriate integration models.

## Supplementary Material

bpaf028_Supplementary_Data
